# Privacy-Preserving Blockchain Technologies

**DOI:** 10.3390/s23167172

**Published:** 2023-08-14

**Authors:** Dalton Cézane Gomes Valadares, Angelo Perkusich, Aldenor Falcão Martins, Mohammed B. M. Kamel, Chris Seline

**Affiliations:** 1 Federal University of Campina Grande, Campina Grande 58429-900, PB, Brazil; perkusic@dee.ufcg.edu.br; 2 Federal Institute of Pernambuco, Caruaru 50740-545, PE, Brazil; 3 Signove Tecnologia S/A, Campina Grande 58400-565, PB, Brazil; aldenor@gmail.com; 4 Department of Computer Algebra, Eotvos Lorand University, 1053 Budapest, Hungary; mkamel@inf.elte.hu; 5 Department of Computer Science, University of Kufa, Najaf 54001, Iraq; 6 Department of Computer Science, Furtwangen University, 78054 Furtwangen im Schwarzwald, Germany; 7 Darkblock, Washington, DC, USA; chris@darkblock.io

**Keywords:** security, trusted execution environments, confidential computing, technical analysis, privacy preservation

## Abstract

The main characteristics of blockchains, such as security and traceability, have enabled their use in many distinct scenarios, such as the rise of new cryptocurrencies and decentralized applications (dApps). However, part of the information exchanged in the typical blockchain is public, which can lead to privacy issues. To avoid or mitigate these issues, some blockchains are applying mechanisms to deal with data privacy. Trusted execution environments, the basis of confidential computing, and secure multi-party computation are two technologies that can be applied in that sense. In this paper, we analyze seven blockchain technologies that apply mechanisms to improve data privacy. We define seven technical questions related to common requirements for decentralized applications and, to answer each question, we review the available documentation and gather information from chat channels. We briefly present each blockchain technology and the answers to each technical question. Finally, we present a table summarizing the information and showing which technologies are more prominent.

## 1. Introduction

A blockchain is a decentralized chain of blocks that register lists of transactions, organizing them hierarchically [1]. Every block added to a blockchain must be mathematically validated by its nodes. This characteristic provides security for the transactions, allowing their auditability. There are three categories of blockchains [2,3]: public, in which anyone can read, send, or receive transactions and any node can participate in the consensus protocol, making decisions regarding the transactions to be accepted; consortium, in which only a set of participants have influence on the consensus process, although anyone can read in the network; and private, in which a unique participant has write permissions and can control the consensus process, although read permissions can be open to anyone or to a set of participants.

Although blockchain technology has gained attention with the rise of Bitcoin [4], many application ideas have emerged since then [5], mainly because of technological advances, such as the adoption of smart contracts, which allow code execution in the blockchain nodes. This evolution runs from Blockchain 1.0 with digital currencies, passing by Blockchain 2.0 with smart contracts, and reaching what we now have: Blockchain 3.0, with a high level of trust, security, and accountability [2,6,7,8,9,10]. Due to the distributed nature of blockchains, many of these new applications are called dApps (decentralized applications). This market is growing fast, with much financial and academic (research) investment.

Generally, a blockchain should ensure the following security characteristics: tamper-resistant, pseudonymity, consistency, and resistance to DDoS (distributed denial-of-service) and double-spending attacks [2]. Although these characteristics provide a good level of security, many applications may demand additional properties. For instance, even with pseudonymity achieved, an adversary can perform de-anonymization inference attacks, gathering user transactions and background knowledge to infer the user’s true identity. Even considering a user can have various pseudonymous addresses, all transactions on the ledger are publicly traceable using the sender’s and recipient’s addresses. In this way, simple analyses can relate the transactions to the used addresses, which can lead to discovering the total amount and number of bitcoins moved to a specific account, for instance. Additionally, it is possible to link multiple accounts that use a unique IP address to sent and received transactions.

Another common characteristic that can be a problem for some blockchains is the lack of confidentiality once addresses and transactions’ contents are available publicly. When considering smart contracts, a requirement is that data and code should be publicly available, which can also be a target for adversaries exploiting these data to infer information from the users.

As we can see, despite the security properties provided by the blockchains, we still have privacy issues because privacy leakage can occur by using publicly available transaction information. Because of this, new blockchains are adopting mechanisms to enhance security and privacy [11]. Besides using specific protocols that require encrypting the sensitive data, some require that part of the blockchain nodes, at least the validators, run on a trusted execution environment (TEE), which executes code in a protected and isolated region of memory. This way, only a TEE application should process the sensitive information. Other blockchains have adopted cryptographic protocols such as secure multi-party computation (SMPC), which distributes the computation of a secret among multiple parties with no party knowing about other parties’ data, and zero-knowledge proof (ZKP), which provides a layer of confidentiality for parties who do not wish to disclose their financial activities publicly.

An example of an application that benefits from these mechanisms is the protection and privacy of authorial content, such as music and images, which need to be stored and negotiated between different parties considering the attribution of NFTs (non-fungible tokens). Thus, a creator may wish to protect the created content and rent or sell it so that only the NFT’s owner can access it (see Darkblock.io (https://www.darkblock.io/)). In addition to protecting the contents, this type of application can also protect the privacy of those who carry out the transactions.

In this vein, we decided to investigate blockchain technologies that deal with data security and privacy concerns and what they propose as solutions. For this, we searched for new blockchain technologies that propose mechanisms to enhance data privacy. After researching, we identified and analyzed the following blockchain technologies: Oasis Network (https://oasisprotocol.org/), Secret Network (https://scrt.network/), Phala Network (https://phala.network), Integritee (https://integritee.network/), Ternoa (https://www.ternoa.com/), NuCypher (https://www.nucypher.com/) [12], and Lit Protocol (https://litprotocol.com/). Then, to analyze each of these technologies, we defined a few technical questions and explored the available documentation (e.g., white papers and websites), chat channels, and news. This paper summarizes the investigated technologies and presents a ranking that considers the answers to the technical questions.

Our main contributions are listed below, considering the seven specified blockchain technologies:

- A brief review of each technology that provides the means to improve data security and privacy;
- A brief analysis regarding each technology based on the specified technical questions;
- A ranking for the technologies considering their technical analyses.

The remainder of this paper is organized as follows: Section 2 presents the basics of blockchains, trusted execution environments, secure multi-party computation, and zero-knowledge proof. Section 3 briefly explains the methodology used for the study, including the definition of the technical questions. Section 4 presents each of the seven blockchain technologies investigated. Section 5 is our technical analysis that considers the answers to the technical questions. Section 6 concludes this work, summarizing the study.

## 2. Background

### 2.1. Blockchain Principles

A blockchain can be viewed as a distributed ordered data structure with a time stamp where only data are appended. Blockchains’ additional properties include immutability, transparency, censorship resistance, and decentralization, enabling a distributed peer-to-peer network. For such a network, non-trusting members can verifiably interact without needing a trusted member [11,13]. Application fields are Internet of Things [14], vehicular networks [15], energy [16], supply chains, transport and logistics [17], and healthcare [18], among many others [5,19]. Blockchain was introduced in the white paper by S. Nakamoto [4] on Bitcoin. It is a distributed ledger that uses independent computers (nodes) to record, share, and synchronize transactions in their respective electronic ledgers, connected in a peer-to-peer network. Transactions are the fundamental units in a blockchain. A definite number of transactions are stored in a block, and blocks are continuously and sequentially appended, resulting in a chain. This highlights the significance of decentralization, where most entities participating in the blockchain are authentic and make the decision collectively based on a consensus mechanism.

Different consensus mechanisms exist [20,21,22], and the one most often used is *proof-of-work (PoW)*. PoW requires solving a complicated computational process, such as finding hashes with specific patterns for authentication and verification. Proof-of-stake (PoS) protocols split stake blocks proportionally to the current wealth of miners [23] instead of splitting blocks proportionally to the relative hash rates of miners, providing a fairer selection mechanism and avoiding the domination of stronger participants. Many blockchains, such as Ethereum in 2022, are gradually shifting to PoS, motivated by the lower power consumption and improved scalability. Byzantine fault tolerance [24] and its variants [5,22,25] are examples of other possible consensus mechanisms.

Blockchain networks can be classified in different ways [5,23,25,26] considering the network’s management and permissions as public, private, and federated or hybrid. New users or node miners can join anytime in public blockchains, also known as *permissionless*. Additionally, participants can perform operations such as transactions or contracts. On the other hand, in private blockchains, where federated belongs to the permissioned blockchain category, a whitelist of allowed users is usually defined with particular characteristics and permissions over the network operations. A critical security aspect is that Sybil attacks are almost impossible there [27,28], so private blockchain networks can avoid expensive PoW mechanisms. Instead, a more comprehensive range of consensus protocols based on disincentives can be adopted. A federated blockchain is a hybrid combination of public and private blockchains [25]. Although it shares similar scalability and privacy protection levels with a private blockchain, the main difference is that a set of nodes, called leader nodes, is selected instead of a single entity to verify the transaction processes. This enables a partially decentralized design where leader nodes can grant permissions to other users. In this work, we provide a more fine-grained blockchain network classification than the current state of the art [13,25,26] because, in addition to classical features such as the ownership and management of the information shared in the blockchain, we consider features such as transaction approval time and security aspects such as anonymity.

More details related to blockchain technology are beyond the scope of this paper. The interested reader may refer to the work of Habid et al. [29] for scalability issues, Hassan et al. [30] for anomaly detection, Ryan et al. [31] and Taylor et al. [3] for security and privacy issues, and Christidis et al. [13] for smart contracts.

### 2.2. Trusted Execution Environments

Trusted execution environments (TEEs) are the basis for confidential computing, which protects data during processing [32]. The TEEs provide a means to create a protected and isolated environment to process data securely, i.e., a TEE creates a tamper-resistant region of memory running with a separated kernel and considering the separation into two execution environments: the “trusted world” and the “normal world” [33]. The isolated and protected environment is the trusted world, which guarantees state integrity for memory and CPU, code authenticity, and confidentiality for data and code.

A TEE application aims to reduce the attack surface, which is limited to the CPU boundary and prevents direct attacks on the sensitive data or code in memory. The idea is that the data enter the trusted world encrypted, are decrypted, and processed securely inside the trusted world, and the results return to the normal world encrypted [33]. The TEE applications maintain data and code confidentiality even if an adversary gets control of the physical machine.

The two more adopted TEE technologies commercially available are the ARM TrustZone (https://developer.arm.com/ip-products/security-ip/trustzone) and the Intel Software Guard Extensions (SGX) (https://software.intel.com/pt-br/sgx). TrustZone requires a trusted operating system to run the trusted world, and SGX works differently, with its trusted environments being called enclaves, which run on the same operating system.

### 2.3. Multi-Party Computation

Multi-party computation (MPC) is a cryptographic protocol that allows a set of parties to perform a distributed function. MPC uses the inputs of the parties in a privacy-preserving manner to execute a function and obtain the results based on those values, without revealing the inputs. Each party encrypts its input and shares the encrypted value with other parties. Therefore, the confidentiality and privacy of the shared values are preserved, and no single party during the process is able to obtain the inputs of other parties. This aspect ensures confidentiality of the inputs, which is crucial in many sensitive domains such as finance [34], healthcare [35], and data sharing between organizations [36]. The parties in MPC then collaborate to jointly obtain the final result, which none of them is able to obtain individually.

As with MPC, blockchain aims to achieve security in a decentralized setting. MPC can be utilized to provide privacy preserving computation in blockchain [37,38]. Additionally, MPC can be implemented to achieve a secure consensus protocol [39] that is distributed and not dependent on a single entity. Therefore, utilizing MPC in blockchain can strengthen privacy and enhance security.

### 2.4. Zero-Knowledge Proof

Zero-knowledge proof (ZKP) is a cryptographic concept that allows one party (a prover) to be able to prove that a statement is true (e.g., knowledge of a particular secret) to another party (a verifier), without revealing any information about the statement except the validity of the statement. The process of ZKP can involve a sequence of interactive interactions between the prover and verifier, in which the prover attempts to authenticate the statement by sharing specific information while maintaining confidentiality and privacy of the statement itself. Based on these interactions, the verifier will be able to securely validate the truthfulness of the claim of the prover without compromising the underlying information. Typically, there are three phases in a ZKP framework [40]. In the witness phase, the prover prepares a proof based on the statement and sends it to the verifier. In the challenge phase, the verifier prepares a challenge based on the received proof and sends it to the prover. In the response phase, the prover solves the challenge and sends the response to the verifier.

ZKP is considered a fundamental tool in modern cryptography and has gained significant attention due to its ability to address critical security and privacy challenges. It can be utilized in many applications, such as digital identity [41], blind signatures [42], attribute verification [43], and key distribution [12]. ZKP offers a powerful cryptographic technique that improves privacy, security, and efficiency in blockchain applications. There are many applications in the implementation of ZKP in blockchain, such as enabling anonymous transactions [44], facilitating secure voting systems [45,46], and supply chain management [47].

## 3. Review Methodology

To carry out this study, we first searched for blockchains that propose security and privacy improvements, applying mechanisms to enhance data confidentiality and privacy. We found the following seven prominent technologies: Integritee, Lit Protocol, NuCypher, Oasis Network, Phala Network, Secret Network, and Ternoa. To guide us in this work, we decided to establish technical questions based on the following principles that we judge relevant for decentralized applications that demand sensitive data protection: security, scalability, cost, interoperability, and support.

For security, we defined three questions considering secure communication, secure processing (confidential computing), and access control. For each of the other principles, we defined one question. For scalability, we refer to the technology’s transaction capacity, e.g., if it successfully processes many transactions simultaneously. For the cost, we want to know the processing charges for the transactions/operations. By interoperability, we mean the capacity to communicate with other blockchain technologies. Lastly, by support, we are interested in a prominent and active community that can provide good documentation and help when necessary. Thus, we defined the following seven technical questions (TQs):

- TQ1—What is the communication with the blockchain nodes? Does it support HTTPS or another secure communication method?
- TQ2—Is it secure? Does it allow/require confidential computing (i.e., trusted processing and storage), MPC, or ZKP? What are the limitations of the programs running in the confidential environment?
- TQ3—Does it have access control mechanisms? What are they?
- TQ4—Does it scale? What is the approximate throughput (requests per day)?
- TQ5—What is the cost? How are payments made? (It is relevant to know what the payment is for the resources consumed.)
- TQ6—Does it support communication with other blockchain technologies? How difficult is the communication?
- TQ7—Is the platform well-supported and well-funded, and does it appear to be successful?

To analyze each technology and answer each of the seven technical questions, we collected information from the official webpages, white papers, news, and chat groups (e.g., Discord (https://discord.com) groups).

## 4. Privacy-Based Blockchains

### 4.1. Secret Network

Secret Network is a blockchain based on Cosmos (https://cosmos.network/) and built with the Cosmos SDK (https://v1.cosmos.network/sdk), which aims to provide privacy, smart contracts, scalability, and interoperability. It employs proof-of-stake using Tendermint’s (https://docs.tendermint.com/master/introduction/what-is-tendermint.html) Byzantine fault-tolerant consensus algorithms and uses CosmWasm (https://cosmwasm.com/) for integration with Cosmos SDK and its ecosystem. The CosmWasm provides secure architecture, tools for developing and testing smart contracts, and Cosmos Inter-Blockchain Communication Protocol (IBC) (https://ibcprotocol.org/) integration. The IBC allows interoperability with other blockchain networks. The native token of the Secret Network is the SCRT, and its smart contracts are called secret contracts (https://docs.scrt.network/dev/secret-contracts.html).

The SNIP-20 (Secret Network Improvement Proposal) (https://github.com/SecretFoundation/SNIPs/blob/master/SNIP-20.md) specifies the interactions among tokens and contracts. It is based on Ethereum’s ERC-20 (https://ethereum.org/en/developers/docs/standards/tokens/erc-20/) and ERC-777 (https://ethereum.org/pt/developers/docs/standards/tokens/erc-777/) standards and is a superset of CosmWasm’s CW-20 (https://docs.cosmwasm.com/cw-plus/0.9.0/cw20/spec/). SecretSCRT (sSCRT) is the first implementation of the SNIP-20 specification and has the following guarantees: all balances and transaction arguments in a transfer are encrypted. Viewing keys can be created to allow third parties or other contracts to access private information (e.g., balance). The token wSCRT is a wrapped SCRT on Ethereum used to provide liquidity and can be redeemed for sSCRT/SCRT (1:1) using the Ethereum–Secret Network bridge (https://bridge.scrt.network/). The secret contracts are Rust-based smart contracts that compile to WebAssembly (wasm). Every six seconds, a block is created and appended to the network, with a limit of 22 transactions per second (however, the theoretical limit is 10,000 transactions per second with the current architecture and protocol) [48].

The network applies encryption protocols, key management, and confidential computing to achieve data privacy. Thus, trusted execution environments (TEEs) are required to protect data processing in all of the network’s validator nodes. Inputs, outputs, and states can be encrypted and securely processed inside a TEE. The consensus seed (256 bits) is the most critical part of the Secret Network encryption schema, being sealed and stored at \$HOME/.sgx\_secrets/consensus\_seed.sealed in the validator nodes. The protocol uses the consensus seed and HKDF-SHA256 (https://www.devglan.com/online-tools/hmac-sha256-online) to derive keys. The public keys are published to the Secret Network genesis.json. Curve25519 (https://cr.yp.to/ecdh.html) is used to generate asymmetric encryption keys, and ECDH (elliptic-curve Diffie–Hellman) is used to derive symmetric encryption keys. These symmetric keys are used to encrypt data with AES-128-SIV.

Secret NFTs, defined by the SNIP-721 (https://github.com/SecretFoundation/SNIPs/blob/master/SNIP-721.md) and based on ERC721 (https://eips.ethereum.org/EIPS/eip-721), allow the NFT owner to decide what data are public and what are private. These NFTs have a name, a value, and a privacy level. The privacy level can be public, protected, or private. For the public level, all the data are publicly available. For the protected level, only some property names are public. For the private level, only the owner can see the name and the value. When an NFT is sold, Stash (https://stashh.io/faq) charges a fee of 2.75% of the NFT price or 0.05 sSCRT, which is automatically deducted by the platform. A small SCRT amount is also required to pay operation fees to the Secret Network. The NFT is encrypted, uploaded to IPFS (https://ipfs.io/), and pinned via Pinata (https://www.pinata.cloud/). Its public preview is available at Azure. A creator can specify a royalty to be deducted automatically by the platform whenever a collector sells the NFT. Although the royalty addresses are private, their percentages and the number of payments are public.

### 4.2. Oasis Network

The Oasis blockchain [49] is a smart contract platform that provides scalability and privacy. Its smart contracts can be efficiently verified and confidentially executed. Oasis was designed to be:

- Flexible—easy to modify system parameters;
- Extensible—easy to add new components like confidential computing techniques;
- Scalable—throughput should increase with the number of nodes;
- Secure—the system should enforce security policies and provide confidential computing;
- Fault-isolated—the system should be fault-tolerant in terms of security and performance.

Oasis’ native token is called ROSE (although the native token of the testnet is TEST). The average time to generate a new block is 6 s.

The platform has a modular design containing two main layers: the consensus layer and the paratimes layer [50]. The name paratimes comes from parallel runtimes, which means multiple runtimes can run simultaneously in the network. A verifiable computing implementation (discrepancy detection) provides an optimized consensus execution that is more efficient than traditional BFT (Byzantine fault tolerance) techniques, improving the smart contracts’ scalability. The consensus layer is based on the Tendermint BFT consensus protocol, uses proof-of-stake as the block proposer protocol, and can be replaced by another consensus mechanism, i.e., allows easy changing of the consensus mechanism. The consensus layer receives a Merkle hash of the encrypted paratime state, keeps the information confidential, and simultaneously supports different smart contract runtimes.

Anyone can implement, register, and operate a paratime. A reference paratime implementation (Oasis Eth/WASI Runtime (https://github.com/oasislabs/oasis-ethwasi-runtime)) enables confidential smart contract execution using TEEs and verifiable computing using discrepancy detection. It supports smart contracts developed in Rust and Solidity. Emerald is the official EVM compatible paratime [51], allowing full EVM compatibility, scalability, 99% lower fees than Ethereum, a cross-chain bridge for interoperability, and easy integration with EVM-based dApps.

The Oasis modular architecture presents a separation between the consensus and paratime smart contract execution layers [52]. It allows enterprises to execute their private paratimes on a specific set of server nodes. If a paratime fails for whatever reason, the others are not affected, and there will be no updates to the blockchain (encapsulation and fault isolation).

The paratimes must pay fees for each consensus layer transaction. A paratime can implement its own token independent of the consensus layer token. The confidential execution of smart contracts occurs within paratimes. For the TEE-based paratimes, the contract executes in a TEE application, and its state is encrypted before being stored. Additionally, such paratimes have a key manager component, responsible for managing the cryptographic keys used by the confidential smart contracts.

Oasis SDK (https://docs.oasis.dev/oasis-sdk/) provides a modular framework to help developers implement paratimes and wasm-based smart contracts using the Rust language. Parcel (https://docs.oasislabs.com/parcel/latest/) is another available paratime, which enables the creation of a secure and privacy-preserving layer for the users’ data. Parcel SDK allows developers to implement access policies, data ownership, governance, and data storage and analyses in a privacy-preserving environment [53]. It has APIs to upload datasets, set policies, provide data consent, register applications for data sharing, and schedule off-chain jobs. Parcel SDK also includes the means to turn any data file into an NFT. The Wormhole (https://wormholenetwork.com/) bridge allows the transfer of Ethereum, Solana, Avalanche, BSC, Terra, or Polygon tokens for the Oasis Network.

### 4.3. Phala

Phala Network (PHA) focuses on secure and private distributed computing [54]. The project started in 2018. Phala is a Web 3.0 computing cloud that supports data privacy while remaining trustless. It offers a service to access distributed computing TEE Secure Enclave through a blockchain [55]. Any participant wishing to purchase secure computer resources and services can do so by acquiring the Phala Network token (PHA) to access it through Polkadot (https://polkadot.network/). The users can access the same services through Polkadot’s Canary Network, Kusama (https://kusama.network/), using PHA or its specific token, K-PHA. The vision is to become the world’s largest P2P computing network, a decentralized cloud based on Web3.

Phala is a Polkadot parachain developed based on the Substrate framework (https://substrate.io/). Thus, it gains access to all of Polkadot’s features, but mainly the relay chain service that provides bridge services to other blockchains. Phala achieves scalability by implementing two software design patterns: event sourcing and CQRS (command query responsibility segregation) (https://www.eventstore.com/cqrs-pattern) [55]. Event sourcing is construed when the events causing state transitions are recorded in an appendonly log instead of storing the latest state of the data. The events receive timestamps and can be re-accessed to rebuild the state at any time. The second software pattern is the CQRS, which handles read/write operations separately. Such engineering decisions are based on the claim that these patterns make the system scale and avoid conflicts.

Interoperability is maintained through secure messaging in contract invocation and token transferring. Phala provides the ability to execute smart contracts by offering [54,55]:

- Confidentiality—only authorized queries to the contract are answered;
- Code Integrity—verification on the blockchain of an output produced by a specific smart contract;
- State Consistency—verification of execution at a specific chain state;
- Availability—no single point of failure (gatekeepers and miners);
- Interoperability—contracts can interoperate with other contracts and blockchains.

The Phala protocol provides the following roles: users, worker nodes, remote attestation service, and blockchain [55]. Users can invoke, query, and deploy smart contracts. Worker nodes run confidential contracts in compatible TEE hardware and are off-chain. Each worker node runs a program called pRuntime, deployed to an enclave, providing a VM to run contracts. There are three types of worker nodes [54]:

- Genesis Node, which bootstraps the network and is destroyed after launch;
- Gatekeepers, which manage the secrets and ensure availability and security of the network;
- Miners, which execute the confidential contracts.

The remote attestation service (RAS) is a public service to validate if a worker node deployed a pRuntime correctly inside a TEE. Phala uses the IAS (Intel Attestation Service) for RAS. The last role is the blockchain, the backbone of the Phala network.

The pRuntime provides, through RAS, the security necessary to execute confidential contracts and implements the Phala protocol. This isolation guarantees that no Byzantine fault can happen unless the pRuntime and TEE are compromised. The executors, or miners, are stateless. They obtain the latest state from a confidential contract by sequentially executing all of the input events on the blockchain or from cached contracts and events after that. The blockchain is the only canonical source of contract inputs. Contract states are encrypted and verified on the blockchain with a symmetric key. Each pRuntime registers its identity and establishes secure connections to users with an asymmetric key pair. Since pRuntime registers on the blockchain, any user can validate its identity. The complete process ensures that all worker nodes need to register on the blockchain before participating in mining or gatekeeper election.

The process to deploy the secure execution of code on a distributed structure is as follows:

- The user/developer publishes the contract to the blockchain;
- Gatekeepers generate a symmetric contract key;
- Gatekeepers save the encrypted key to the blockchain;
- The user/developer finds an available miner to load the contract;
- The miner pRuntime connects to a gatekeeper through a secure connection and asks for the contract key;
- The miner uses the received key to encrypt the contract state and saves it to the blockchain.

### 4.4. Integritee

Integritee is the new name for a parachain from a W3 foundation grant called Substratee, having the objective to provide a trusted off-chain compute framework for substrate blockchains (SubstraTEE GitHub) (https://github.com/integritee-network/substraTEE).

It is now under a company called Integritee AG (https://integritee.network/company), which is responsible for driving the development and community efforts for Integritee. The network launched its token TEER to cover payment and governance [56]. Until recently, it has operated its mainnet as an independent project. In February 2022, the community successfully secured a parachain slot in Kusama through a crowd loan process, where they provided rewards for those earlier investors. Their book (Integritee Book) [57] defined Integritee as a framework for parity substrate, allowing it to call a custom state transition function (STF) inside a TEE, namely an Intel SGX enclave, thereby providing confidentiality and integrity. The enclaves operate in an encrypted state that can be read and written to only by a set of provisioned and remote-attested enclaves.

The Integritee website states that the community aims to be the blockchain choice for a secure operating environment that will be scalable, decentralized, and trusted. The company behind Integritee declares that they can scale up to 1 M transactions per second (TPS) due to the decentralized choice of using Polkadot and Kusama infrastructure. The tokenomics of Integritee provide a cap on the token availability of 10 M TEER for the project [58].

According to developers, Integritee can provide:

- Confidential decentralized state transition functions for private transactions, private smart contracts, off-chain confidential personal data records (GDPR), decentralized identity with selective disclosure, and subscription-based content delivery networks;
- Scalability by providing a second layer to substrate-based blockchains for off-chain smart contracts and payment hubs;
- Trusted chain bridges;
- Trusted oracles.

According to Integritee GitHub [59], the project is structured as:

- The Substratee node (archived);
- Integritee Node (Substratee node with TEE registry validating remote attestation);
- Integritee Worker (Integritee off-chain worker and sidechain “validateer”).

An example of an appropriate use case would be a content delivery network (CDN) [60]:

1. Subscriptions managed on-chain, and an Integritee worker holds the content-encryption key (CEK—RSA-AES) to IPFS and registers the content on-chain;
2. Consumers request content from the Integritee worker over a TLS channel (e.g., HTTPS or WSS), and the worker authenticates the consumers and looks at subscription status on-chain;
3. Fetches the trusted content from IPFS;
4. Decrypts the content;
5. Sends the content to the consumer over the previous TLS channel.

The Integritee project completed the M6 and M7 milestones, providing modularity in RUST and the possibility to create shards distributed under multiple processors (Intel SGX). A new feature (not yet funded) for the subsequent releases is the possibility of supporting Ink! (https://ink.substrate.io/) (Substrate’s smart contracts language). This feature is a crucial functionality for enabling ease in implementing the support for NFT handling inside the platform.

### 4.5. Ternoa

Ternoa’s founder created this blockchain to share his memories with his children in the future. The NFTs work as the vehicle for data transmission and data handling. Ternoa provides the means to store data permanently in any format, with the user controlling access and availability [61]. CAPS is the token of the Ternoa blockchain used for payment and governance of transactions.

Ternoa allows secure data storage and transmission, providing an SDK to help develop and integrate applications [61]. It is based on the Substrate (https://substrate.io/) framework and the Polkadot blockchain, and it is designed to be a parachain of Polkadot. Thus, Ternoa enables the connection to other Polkadot-based blockchains. For decentralized storage, it uses other blockchains such as Storj (https://www.storj.io/), Sia (https://sia.tech/), or Arweave (https://www.arweave.org/). The Rust language is used to develop the smart contracts, and the NFTs are based on the ERC721 standard.

As Ternoa is a Polkadot-based blockchain, the community claims that it consumes approximately 0.001% of the Bitcoin blockchain’s energy consumption. This reduced consumption is possible because the Ternoa blockchain uses proof-of-stake (NPOS—nominated proof-of-stake) as the block proposer protocol instead of proof-of-work (PoW) [62]. The PoW consumption is estimated at 48.14 kWh per transaction, while the NPoS consumption is estimated in 0.8 GWh per year (800,000 kWh) [63].

Regarding security, data are encrypted and sent to decentralized servers. The scheme uses a Merkle tree for each stored file. Ternoa has a social recovery module called Trusted Friend [61], which allows users to recover accounts in case of losing the authentication key. The user needs to choose M of N “trusted friends” to enable the process of account recovery. To benefit from the social module, a user must hold encrypted keys from other users.

The Ternoa chain has the concept of capsules [64], which encapsulate the encrypted data and are associated with NFTs. Each capsule contains a unique share. This share can be encrypted and stored on different cloud services and decrypted by the NFT owner. The encrypted share can be exported in text format (txt). Ternoa uses Shamir’s secret sharing (https://medium.com/@keylesstech/a-beginners-guide-to-shamir-s-secret-sharing-e864efbf3648) (SSS) to secure the capsules, splitting the sensitive data into multiple “shares” used to reconstruct the original data [65]. A threshold defines the minimum number of shares needed to rebuild the data. The capsules keep data protected by using asymmetric GPG encryption (https://www.redhat.com/sysadmin/encryption-decryption-gpg).

The basic communication flow follows these steps:

1. Create a capsule with an NFT;
2. Encrypt the capsule content with a GPG key;
3. Generate shares from the GPG key using the Shamir secret sharing method;
4. Send the shares to master nodes with Intel SGX;
5. Define the time protocol for the capsule and send it to the Ternoa chain.

The time protocol specifies when a capsule should be delivered. When the time protocol is triggered, the recipients can retrieve the capsule and claim the shares to obtain the GPG key and decrypt the capsule’s content. A transfer protocol allows for sending the capsule’s keys to a new owner based on a specific date. The blockchain has specific protocols to pass a capsule’s access to other users according to conditions such as the owner’s death or a specified date/countdown. Currently, there is no exact price for the capsules, but the costs will depend on the capsule’s design, the transmission date, and the weight of the transmitted files.

The mainnet was planned for Q1 2022 [66]. The testnet has over 200,000 minted NFTs, 150 nodes installed worldwide, and more than 35 marketplaces [67]. According to DotMarketCap (https://www.dotmarketcap.com/), Ternoa is 31st among the most active Polkadot-based blockchains in terms of capitalization, with a market cap of almost USD 30 M. There is a bridge for exchanging Ethereum (ERC20) and Binance (BEP20) tokens [68].

### 4.6. NuCypher

NuCypher [69] is a data encryption and protection layer for Ethereum (and eventually other public networks) and decentralized applications (dApps) that do no rely on a central service provider. The protocol, which the team calls a decentralized key management system (KMS), allows developers to store, share, and manage private data on public blockchains. Developers receive this encryption service via a network of NuCypher nodes in exchange for a fee (paid for in ETH). Participants can only spin up a node by staking NuCypher’s token, NU, on the network as collateral.

NuCypher is a blockchain-based cryptographic infrastructure for privacy-preserving applications, dynamic control access, secrets management, and secure computation [69,70]. Additionally, NuCypher enables users to manage a range of computational secrets, such as identity and access management (IAM) tokens and database and secure shell (SSH) credentials to access servers remotely.

NuCypher uses a decentralized network to remove the dependency on central service providers, to create a proxy re-encryption for cryptographic access control, and to generate a token incentive mechanism to ensure reliability, availability, and correctness [71,72]. Because of proxy re-encryption, an unencrypted symmetric key that can decrypt private data is never exposed server-side. There is no single point of security failure. Even if compromised, hackers would only obtain re-encryption keys, as access to the file is still protected.

The technology provides a decentralized key management system based on blockchain technology and claims it can be used in DDRM, decentralized digital rights management, for secret key transformation [72]. In NuCypher’s network, the content key is encrypted by the owner’s public key, and only the owner’s private key can decrypt it. With authorization from the owner, the encrypted key will be fragmented and re-encrypted by several proxy nodes. Nodes are unaware of each other and cannot collude with the receiver. After re-encryption, the receiver collects the re-encrypted fragments and decrypts them.

The NuCypher network focuses on providing extensible runtimes and interfaces for data, called secrets, management, and dynamic access control. It provides shared access to data based on a proxy re-encryption schema (PRE). Access permissions are baked into the underlying encryption, and the data owner is the only one who can explicitly grant access via sharing policies [72]. Consequently, the data owner has ultimate control over access to their data. The NuCypher network cannot decrypt the data nor determine the underlying private keys. NuCypher KMS is a decentralized key management service and cryptographic access control layer for the blockchain and decentralized applications. Developers and enterprises can leverage it to create highly secure applications in healthcare, financial services, and more. By bringing private data sharing and computation to the public blockchain, NuCypher KMS enables everything from encrypted content marketplaces to secret credentials management and patient-controlled electronic health records.

### 4.7. Lit Protocol

The Lit (lockable interactive token) Protocol is a decentralized access control protocol running on top of Ethereum and other Ethereum virtual machine (EVM) chains (full-list EVM chains) (https://github.com/LIT-Protocol/lit-js-sdk/blob/main/src/lib/constants.js#L14). Lit on-chain access control conditions allow for [73]:

- Encrypting and locking static content among images, videos, and music behind an on-chain condition such as ownership of an NFT;
- Decrypting static content that was locked behind an on-chain condition;
- Authorizing network signatures that provide access to dynamic content (for example, a server or network resource) behind an on-chain condition;
- Requesting a network-signed JWT (JSON web token authentication) that provisions access and authorization to dynamic content behind an on-chain condition.

With this functionality, the Lit protocol enables the creation of locked NFTs that only their owners can unlock [74]. It also allows access to a given server or network resource only to NFT owners. Rather than a simple JPEG, Lit NFTs can be HTML/JS/CSS webpages that can be interactive and dynamic.

The network acts as a decentralized access control list (ACL) that leverages on-chain data to grant users access to content, software, and other decentralized networks [75]. Lit supports many standard contracts and plans to support any RPC call soon. The Lit Protocol is in an alpha state (the “AlphaNet”), and the creators are running all the nodes. During the writing of this paper, the Lit network was unaudited, and the nodes still need to be distributed. Various security improvements must be made, and crypto-economic guarantees resulting from staking are not yet in place. Data are persistent and planned to perpetuate the network. Data can be stored, for example, in IPFS and Google Drives. Developers can use the Lit Protocol SDK [76], which is currently integrated with EVM chains and storage providers like Ceramic Network (https://developers.ceramic.network/learn/welcome/).

For static content, the SDK encrypts the user’s content and uploads the conditions for decryption to each Lit Protocol node. When someone wants to access the content, the SDK requests a message signature from the user’s wallet that proves the user owns the NFT associated with the content to each Lit Protocol node. The Lit Protocol nodes will then send down the decryption shares, and the SDK will combine them and decrypt the content.

The SDK can create the authorization conditions for a given resource and store them with Lit Protocol nodes for dynamic content. For this type of content, the flow is similar: when an entity requests a network signature to access a resource—typically a server that serves some dynamic content—the SDK also requests a message signature from the wallet and verifies if the entity owns the NFT associated with the resource to each Lit Protocol node. Each node will verify what entity owns the NFT, sign the JWT to create a signature share, and then send down that share. The SDK will combine the signature shares to obtain a signed JWT, which can be presented to the resource to authenticate and authorize the user.

Nodes can provide the user a key to access specific content, whether to decrypt something or access some service. That is a gateway that many can use. For instance, Shopify (https://www.shopify.com/) merchants could use it to enable NFTs to act as coupon discounts. The nodes can also provide conditions for unlocking; for example, someone who owns more than three CryptoPunks (https://www.larvalabs.com/cryptopunks) can access a given file. A user sends signed messages to each blockchain node to unlock something, creating a decryption share. The network uses a BLS (Boneh–Lynn–Shacham) threshold encryption (https://alinush.github.io/2020/03/12/scalable-bls-threshold-signatures.html). Then, like a torrent, those decryption shares are sent to the user in the client.

Encrypted messages, where only the user’s address can decrypt a message, can also be used. The net result is that the user data are sovereign. Users can allow various individuals, applications, or agents to access those data. That means one can own the data and decide what to do with them on the decentralized web without relying on centralized authorities to hold the data. The protocol enables on-chain conditions, such as NFTs, to act as keys to Web2 and Web3 experiences. There is no limit to the amount of data a token can be used to control access to because it is up to the user to decide where they want data stored. The Lit Protocol provides the access control layer in the stack. Additionally, the user might not constantly be unlocking data and could be opening a perk, reward, content, or metaverse experience.

There are multiple parties in the network. Some nodes provide the service, with different parties performing encryption and decryption. Whether this is legal or not in a particular country varies with each nation’s encryption laws and policies. It depends on the rules in a stakeholder’s role and their location.

Also, there is a portal for connecting blockchain wallets to the rest of the internet, powered by a Lit Protocol called Lit Gateway [77]. Apps let a user create resources exclusive to a crypto community, for example, Google Drive files that are only accessible to members of the user’s DAO (decentralized autonomous organization) or a given NFT’s owners. It can offer rewards, discounts, NFTs, and airdrops that can only be accessed if the wallet meets specific criteria, such as owning a given token. Once a wallet is connected, offers that are available can be seen.

### 4.8. Summary

**Figure 1:**
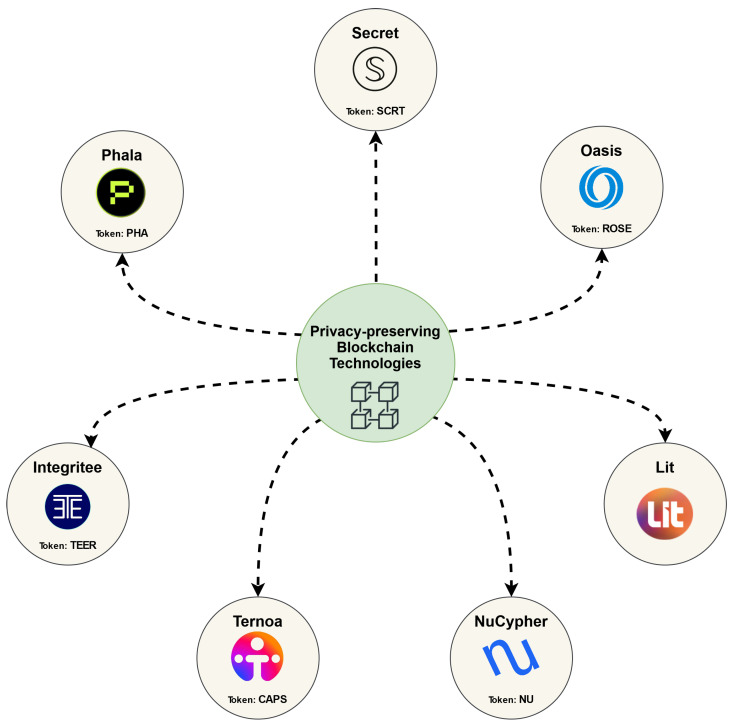
Privacy-preserving blockchains.

As shown in Figure 1, we have discussed seven privacy-preserving blockchain technologies. Table 1 summarizes the properties of the discussed technologies in this section, in terms of the blockchain basis, token name, TEE support, MPC, and ZKP.

## 5. Technical Analysis

In this section, we answer each of the seven technical questions described in Section 3 for each of the technologies described in the previous section.

### 5.1. What Is the Communication with the Blockchain Nodes? Does It Support HTTPS or Another Secure Communication Method?

#### 5.1.1. Secret Network

In general, developers use SecretJS (https://www.npmjs.com/package/secretjs) to connect to a Secret Network node. SecretJS is a JavaScript/typescript library based on the CosmWasmJS (https://github.com/CosmWasm/CosmWasmJS) library, which allows for the creation of a client that connects to a node. The available examples use an HTTPS address for communication with the network nodes, but it is unclear if the communication is performed only with HTTPS. Independently, the communication channel is only one of the concerns regarding data transmission, which can be mitigated by encrypting the sensitive data before transmission. This way, a protected communication channel (HTTPS, for instance) is a plus, not necessarily a requirement.

#### 5.1.2. Oasis Network

Yes, the blockchain supports secure communication with the nodes. By looking at a few code examples, we can see that communication can be established with HTTPS or WSS.

#### 5.1.3. Phala Network

All the worker nodes are non-Byzantine nodes, and all contract communications are encrypted off-chain. All communication was designed to be secure using asymmetric, symmetric keys, key rotation, node registration, state recovery, and monitoring of responsiveness, in which case the non-compliant nodes will be slashed when the gatekeepers are under more severe scrutiny. Looking at some code examples, we can see that the communications are established with HTTPS or WSS.

#### 5.1.4. Integritee

Integritee will work on top of Polkadot and Kusama. Depending on the application, the project allows for specifying what will be executed securely on TEE and what parts will be processed on- and off-chain. All Polkadot/Kusama relay/parachains can interface with Integritee workers or sidechains through the XCMP (Polkadot cross-chain messaging protocol). The communication of off-chain workers can happen over any TLS channel.

#### 5.1.5. Ternoa

In general, developers use the SDKs provided by the community. In Ternoa’s GitHub, we can find SDKs for the testnet operations, SecretNFTs (https://www.secret-nft.com/), and marketplace. The documentation is not very good or clear, revealing only the API endpoints. We deduce that the Rust language is used for the smart contracts’ development, while NodeJS is used for the dApps development. In the code examples, we can see URLs with HTTP and HTTPS. We are unsure if they require HTTPS in the testnet, but we believe the mainnet will require it. Independently, the communication channel is only one of the concerns regarding data transmission, which can be mitigated by encrypting the sensitive data before transmission, as mentioned before.

#### 5.1.6. NuCypher

According to the NuCypher blog, the underlying threshold PRE scheme used in NuCypher, called Umbral, has been rewritten in Rust (rust-umbral), which then can be compiled into JavaScript. Additionally, NuCypher itself is being rewritten in TypeScript (nucypher-ts (https://github.com/nucypher/nucypher-ts)). It is still ongoing work and will allow developers to build apps with full PRE functionality (grant, receive, revoke, among other operations). NuCypher also provides REST-like HTTP endpoints for working with characters (HTTP character control).

#### 5.1.7. Lit Protocol

The SDK requires an active connection to the Lit nodes to perform most functions (notably, a connection to the Lit nodes is optional if you are verifying a JWT). The connection is typically done on the first page load in web apps and can be shared between all its pages. In NodeJS apps, this is done when the server starts. Additionally, a web-ready package is provided, with all dependencies included, at build/index.web.js, which can be imported to a webpage using a script tag:

$<scriptonload{=}^{\prime }litJsSdkLoaded{\left(\right)}^{\prime }src=“https://jscdn.litgateway.com/index.web.js^{\prime \prime }></script>$

### 5.2. Is It Secure? Does It Allow/Require Confidential Computing, MPC, or ZKP? What Are the Limitations of the Programs Running in the Confidential Environment?

#### 5.2.1. Secret Network

Yes, it requires that the validator nodes run on TEEs, which run code securely even if an attacker has privileged permissions on the node host. Although TEE is not a “bullet-proof” security solution, it is the most secure one that is currently employed. Thus, the validator nodes run secret contracts preserving data privacy during processing. The TEEs in the validator nodes, together with the encryption mechanisms and the Secret Network standards (SNIP-20 and SNIP-721), provide privacy by design to the network. Intel SGX is the TEE in use for the current validator nodes. Thus, the limitations are the memory size limit for running code and the programming difficulty, which is considered challenging by the community. The memory size should be fine for the secret contracts unless they run complex operations demanding a high amount of memory. Regarding the programming difficulty, code libraries may arise to help the development of secret contracts.

#### 5.2.2. Oasis Network

Yes, the confidential paratime called Oasis Eth/WASI Runtime (https://github.com/oasislabs/oasis-ethwasi-runtime), also called Cipher, allows for running smart contracts that preserve data privacy since the paratime’s nodes employ TEE. This way, the smart contracts receive encrypted data, decrypt and process them inside a protected memory region (e.g., enclave), and encrypt the results before transmission. As the employed TEE is Intel SGX, the limitations are the same for the Secret Network. Regarding programming difficulty, the Oasis community provides SDKs to ease the development of dApps and smart contracts using Rust and Typescript languages.

Another paratime called Parcel employs GCP (Google Cloud Platform) confidential VMs, which provide VMs that run on AMD SEV processors. The Oasis documentation suggests the development of other confidential paratimes using other security mechanisms, such as homomorphic encryption, zero-knowledge proof, and secure multi-party computation.

#### 5.2.3. Phala Network

All the operations are under a confidential contract and, by definition, end-to-end encrypted between TEE, blockchain, and the user. The communication between TEE and the user is end-to-end encrypted with the Diffie–Hellman algorithm. Developers can implement “fat contracts” using the Rust language.

#### 5.2.4. Integritee

Yes, Integritee allows secure data processing by requiring/providing Intel SGX (TEE) in their nodes. The overall security depends on the application design that will use Integritee. For example, a project can be designed to run a CDN where the keys to decrypt the content are the only data processed under the TEE. Once the process is confirmed, the keys to decrypt will be provided to the client and pointed to the URL holding the content. To attest that a specific node runs on an Intel SGX enclave, the requester node checks the remote enclave’s information with the IAS (Intel Attestation Service).

By design, Integritee can adapt to many use cases. The developer decides which part should work under the TEE.

#### 5.2.5. Ternoa

Although there is no good documentation, we deduce that the blockchain requires that the validator nodes, called masternodes, run on trusted execution environments (TEEs). The TEEs in the masternodes and the encryption mechanisms provide privacy by design to the network. Intel SGX is the TEE in use for the current masternodes, although someone said in the Discord channel that they were changing: “Hi, at the beginning we were planning to use SGX from Intel, but we had to change the technology. Some node types use the TEE to encrypt the information and make sure. There will be few types of nodes, and not all of them will have the TEE features”.

We did not receive answers when we asked for more information regarding these TEE nodes and their operations. Thus, the limitations are the memory size limit for running code in the SGX enclaves (if the nodes are still using Intel SGX). Regarding the programming difficulty, we believe the Ternoa chain is using the Rust language to develop SGX applications.

#### 5.2.6. NuCypher

It is secure from the application point of view through the use of PRE. There is no need for confidential computing specific hardware support such as TEE.

#### 5.2.7. Lit Protocol

Yes, it uses BLS threshold encryption. However, it does not protect data processing, i.e., there is no need for confidential computing-specific hardware support such as TEE.

### 5.3. Does It Have Access Control Mechanisms? What Are They?

#### 5.3.1. Secret Network

Yes, but it is not fine-grained. The private information is only available for its owner or those who receive the viewing keys. Thus, access control relies on the viewing keys controlling who can see the private information. Regarding the network nodes, the protocol requires that the validator node candidates run on Intel SGX (TEE) and follow some specific rules.

#### 5.3.2. Oasis Network

Yes, at least for the Parcel paratime. According to its documentation, an entity can establish grants (policies) that allow other entities to access data. The Parcel SDK provides APIs to set policies and manage permissions.

#### 5.3.3. Phala Network

The Phala Network provides three kinds of entities: client (user), which operates on normal devices with no need for special hardware; worker, which operates on the TEE and computes confidential contracts; gatekeeper, which operates on the TEE and serves as the authority and key manager. There is no information regarding fine-grained access control mechanisms for dApps. Thus, the dApps should implement their permissions protocol.

#### 5.3.4. Integritee

Integritee blockchain does not have native and fine-grained access control mechanisms. Thus, the applications must implement access control. As mentioned earlier, the client will provide the TEE worker with what should be processed under the Integritee chain and what should be processed off-chain. The communication channels can be encrypted over TLS. The TEE environment has security guarantees provided by Intel SGX and can be attested with the Intel Attestation Service (IAS). The public attestation part of the task’s action would be registered over the Integritee blockchain, providing a public audit.

#### 5.3.5. Ternoa

Yes, but it is not fine-grained. The private information is only available to its owner or those who receive permission to access the capsules’ content through the available protocols. Thus, access control relies on the NFT owners controlling who can see the private information. The protocol requires that the masternodes run on TEE and follow some specific rules regarding the network nodes.

#### 5.3.6. NuCypher

Nucypher implements dynamic access control that conditionally grants and revokes access to sensitive data. Conditional access specifies conditions for sharing data, such as time-based and behavior-based access. Access revocation revokes access on demand or automatically under customizable, pre-specified conditions.

#### 5.3.7. Lit Protocol

Yes, access control conditions are based on standard contract types like ERC20, ERC721, and ERC1155. Additional conditions are wallet address ownership, proof of humanity, and possession of POAP (Proof of Attendance Protocol). Conditions can be set and define how to grant access.

### 5.4. Does It Scale? What Is the Approximate Throughput?

#### 5.4.1. Secret Network

Yes, the community claims the network is scalable, but it is unclear how scalable. The gray paper mentions that the theoretical cap is 10,000 transactions per second, which results in 864,000,000 transactions per day.

#### 5.4.2. Oasis Network

Yes, the community claims the network is scalable and versatile. The Emerald parachain allows a throughput of 1000 TPS, which gives 86,400,000 transactions per day.

#### 5.4.3. Phala Network

Due to protocol decisions, they were able to minimize duplication of execution for validation and to decouple the execution from consensus tasks, as the most intensive tasks inside the TEE are executed off-chain. They have already achieved a trustless cloud of 20,000 registered computing nodes (workers), with 15,000 running on Khala. These servers provide around 120,000 vCPUs. The project states that Phala can manage as many as 1 million CPU cores from over 100,000 nodes.

We did not find any information regarding the Phala throughput; however, as Phala is a parachain of Polkadot, we believe its scalability is similar to that of Polkadot, which is 1000 TPS (86,400,000 transactions per day). However, the community claims it could reach a greater number when the network evolves to its full operation.

#### 5.4.4. Integritee

The project states that the Integritee blockchain will hold up to 1 M TPS, the claimed maximum throughput for Polkadot-based blockchains (parachains). There is a limitation regarding Polkadot/Kusama’s capacity to handle over 100 parachains. Securing a parachain is accomplished through a bid process where different projects compete for a slot, which can become pricey competition.

#### 5.4.5. Ternoa

Since the documentation does not mention information regarding the blockchain’s scalability, we asked in the Discord channel. However, we did not receive the information: “Currently, we have no fully updated doc as many things have evolved since the beginning of the blockchain building. However, the team knows that it’s necessary and is working on creating the documentation and then updating the white paper with the new core product/feature”.

As Ternoa was developed to be a parachain of Polkadot, we believe its scalability is similar to Polkadot’s scalability. Additionally, the Polkadot community claims the parachains can improve throughput and scalability. For now, we underestand that Polkadot is offering 1000 TPS (86,400,000 transactions per day), but this number could reach 166,000 (or even 1,000,000 when the network evolves to its full operation).

#### 5.4.6. NuCypher

NuCypher’s decentralized access control system offers developers and their users a departure from this opaque and trust-dependent paradigm. It enables end-to-end encrypted data-sharing workflows within applications without sacrificing scalability, redundancy, or performance. It is applicable to data payloads of any form, size, structure, sensitivity, or production cadence. Users share privileges they currently take for granted but are not obliged to trust the application developers or third-party access control services, such as centralized servers or key management systems, with their data.

#### 5.4.7. Lit Protocol

It is not available in the documentation. We asked on Discord about details but did not receive an answer. Nevertheless, as the Lit Protocol runs on top of Ethereum and this blockchain is still moving the block proposer protocol from proof-of-work (PoW) to proof-of-stake (PoS), we can associate the scalability of Lit Protocol with that of Ethereum: 12–15 transactions per second (TPS). This scalability gives more than 1 million transactions per day. When Ethereum 2 starts, the community claims its scalability could reach 100,000 TPS.

### 5.5. What Is the Cost? How Are Payments Made?

#### 5.5.1. Secret Network

The payments are made in SCRT, the native token, but depend on the operations performed. The launched Supernova mainnet has promised fees up to 10 times cheaper. In general, it seems the minimum gas fee is SCRT 0.25. According to a contract’s sample code, the following are some suggested fees for the main operations: “upload”—SCRT 5, “init” and “exec”—SCRT 0.5, “send”—SCRT 0.08.

#### 5.5.2. Oasis Network

The payments are made in ROSE, the platform’s native token, but depend on the operations performed. The community claims Oasis has 99% reduced fees compared to Ethereum. In the Oasis Discord channel, we received the information that the transactions “usually land at about 0.000001 Rose or roughly 2.6 × 10${}^{-7}$ USD for a regular transfer”.

#### 5.5.3. Phala Network

There are already PHA 272,000,000 in circulation, with a maximum supply of 1,000,000,000, and it is already traded in Binance, OKX, DigiFinex, Mandala Exchange, and CoinTiger, where the current value of the token (as of 2 February 2023) is USD 0.1824.

Although we did not find information regarding the cost of operations, we believe the payments are similar to Polkadot, which adopts a weight-based fee model instead of a gas-metering model. The fees are calculated based on these three parameters: weight fee (base + calls weight), length fee, and an optional tip. The weight fee is based on the time spent to execute the transaction, and the length fee is a multiplier applied to the transaction’s size (in bytes).

#### 5.5.4. Integritee

Integritee has a governance council that will decide the price to operate in the blockchain. All the work will be rewarded as a TEER token. The token has the dual function of utility and governance. The TEER used as a utility will reward those tasks inside the chain. The TEER used in the governance works as a stake to operate the Integritee blockchain, promote development and counsel, and decide the price that will operate in the TEE oracle to USD. The Integritee project plans to have companies participating as providers for those services (fiat, TEE), although Integritee AG is the only one operating in the ecosystem.

We believe the payments will be similar to Polkadot, so the same explanation as for the Phala Network applies to Integritee (i.e., the fees consider the mentioned parameters).

#### 5.5.5. Ternoa

We did not find information regarding costs in Ternoa’s documentation, even when we asked on the Discord channel. We could estimate as we did for scalability, based on Polkadot, but we read that the parachains do not depend on the Polkadot fees. They are independent and require their specific taxes in their specific tokens. As we believe the payments are similar to Polkadot, they also follow what we explained for the Phala Network.

#### 5.5.6. NuCypher

The minimum and default fee rates are GWEI 350, while the maximum fee rate is GWEI 3500 per period, per policy, per Ursula. An Ursula is a node that receives information about a user policy to access encrypted data and is rewarded for re-encrypting those data through proxy re-encryption. Ursulas are to NuCypher what validators are to other proof-of-stake networks.

The minimum and maximum fee rates are the lower and upper bounds that constrain the fee rate a “staker” may offer. The default fee rate is the rate that will be displayed and provided for Alices if the staker chooses not to configure this parameter themselves or chooses a rate outside the boundaries of the global fee range. The default rate will also be used if the range’s boundaries are updated, if a staker’s specified rate now falls outside the range, and if they fail to change it.

#### 5.5.7. Lit Protocol

It is not available anywhere in the documentation. We asked on Discord about details, but we did not receive an answer. Gas or transaction fees are network-dependent, and Lit can interact with any EVM-based chain. It should follow the basics of any EVM-based network.

### 5.6. Does It Support Communication with Other Blockchain Web Technologies? How Difficult Is the Communication?

#### 5.6.1. Secret Network

Yes, there are already some bridges between distinct blockchains (e.g., Ethereum, Binance, and Monero) and the Secret Network, and they are implementing solutions based on IBC (Inter-Blockchain Communication). The Secret Network already participates in the “IBC Gang”, together with other blockchains.

#### 5.6.2. Oasis Network

Yes, there is a bridge between distinct blockchains (e.g., Ethereum, Solana, Avalanche, BSC, Terra, and Polygon) and the Oasis Network. Moreover, the Oasis Network paper mentions that the IBC protocol can be applied for communications between paratimes.

#### 5.6.3. Phala Network

Yes, Phala is built as a Polkadot parachain and can benefit from Polkadot’s shared security, transaction settlements, and consensus. A registered user in the Polkadot ecosystem requests a quote through a gatekeeper to execute a smart contract following the Phala protocol. By definition, any blockchain in Polkadot can access a TEE through the Phala blockchain.

#### 5.6.4. Integritee

Most of the project is written using Substrate in Rust, a framework with the main goal of providing chains of chains. The system supports Polkadot and Kusama, and, by design, Polkadot can interface with different chains. In the light paper [78], the authors describe their intent to support the Ethereum blockchain.

#### 5.6.5. Ternoa

Yes, currently, there are bridges for Ethereum and Binance blockchains. Additionally, communication with Polkadot and any of its parachains is considered simple.

#### 5.6.6. NuCypher

Yes, it is a decentralized threshold cryptography service implemented as a layer 2 network on top of Ethereum.

#### 5.6.7. Lit Protocol

Yes, the Lit Protocol is a decentralized access control protocol running on top of Ethereum and other Ethereum virtual machine (EVM) chains (full list of EVM chains): Ethereum; Polygon; Fantom; Xdai; Bsc; Arbitrum; Avalanche; Harmony; Kovan; Mumbai; Goerli; Ropsten; and Rinkeby.

### 5.7. Is the Platform Well-Supported and Well-Funded? Does It Appear to Be Successful?

#### 5.7.1. Secret Network

Yes, the community is growing, attracting investments. In 2022, they announced an investment of USD 400 million to be applied in the Shockwave, their new initiative to solidify the network as the Web3 privacy hub.

#### 5.7.2. Oasis Network

Yes, the community has attracted investments. In 2022, the platform received financial support from Binance, reaching USD 200 million for expanding the Oasis ecosystem. Additionally, companies such as Meta AI are becoming Oasis’ partners.

An interesting application built on Oasis is The Music Fund (https://themusic.fund/), which gives funds to artists in advance for a percentage of royalties over two years.

#### 5.7.3. Phala Network

Yes, the platform was well-funded by IOSG Ventures in September 2020. The company was founded in 2018, with one round, not publicly disclosed, and is at the seed stage. Phala raised USD 1.68 M in token sales in two rounds. In 2022, it joined the Blender Developer Fund to accelerate Metaverse 3D Modeling and Rendering. The company received three Web3 Foundation grants. Khala Network is the Phala activity inside the Kusama blockchain, where they secured a slot in July of 2021. The company secured a Kusama slot, raising KSM 132,281 (USD 15,258,584). Phala Network is the activity of Phala inside Polkadot. They raised 343,024 DOT (USD 5,460,949) in 2021.

#### 5.7.4. Integritee

The platform is funded with grants from Web3 and a total investment of up to USD 6.5 M. In 2022, the community secured a Parachain in Kusama through Crowdloan (https://polkadot.network/features/crowdloans/), a proof of confidence from the crypto market in the proposal. The project secured a slot in Kusama in February of 2022, raising a total of KSM 20,000 (USD 2,298,200).

#### 5.7.5. Ternoa

Not so much; the documentation could be improved a lot. Although the mainnet was launched in the first half of 2022, we could not find detailed information regarding the blockchain or its dApps (e.g., SecretNFT).

#### 5.7.6. NuCypher

Most information on the use is from the academic side.

#### 5.7.7. Lit Protocol

Documentation is quite limited [76]. Lit Protocol has few followers. Discussion on the Discord channel is superficial and not quite technical. However, the community raised USD 2.2 M to use NFTs for decentralized access passes.

### 5.8. Summary

Table 2 summarizes the investigated technologies, considering each technical question. For each question, we evaluated the technology with a number in the range of 1–5, with 1 meaning not available and 5 meaning excellent. We added an extra column regarding the SDKs and tutorials available for each investigated technology.

## 6. Conclusions

Although the general blockchains grant some security properties, they lack mechanisms to protect the privacy and confidentiality of sensitive data. To solve this issue, new blockchain technologies are applying techniques, tools, and protocols such as TEE, ZKP, and MPC. In this context, we have searched the available technologies and analyzed how prominent they are, considering what they propose to enhance data confidentiality and privacy. For this analysis, we defined seven technical questions based on the following five principles: security, scalability, cost, interoperability, and support. We then analyzed seven blockchain technologies, summarizing their strengths and weaknesses, and classified them considering the answers to the technical questions.

For future work, we suggest running basic experiments with the seven investigated technologies, producing a benchmark and a testbed. Additionally, information on other new technologies can enrich our study.

## Figures and Tables

**Table 1:** Comparison among the privacy-preserving blockchain technologies.

Technology	Blockchain Basis	Token Name	TEE	MPC	ZKP
**Secret**	Cosmos	SCRT	✓	✗	✗
**Oasis**	Ethereum	ROSE	✓	✗	✗
**Phala**	Polkadot	PHA	✓	✗	✗
**Integritee**	Polkadot	TEER	✓	✗	✗
**Ternoa**	Polkadot	CAPS	✓	✗	✗
**NuCypher**	Ethereum	NU	✗	✗	✗
**Lit Protocol**	Ethereum	-	✗	✗	✗

**Table 2:** Summary.

Technology	Secure Channel	TEE on Nodes	Access Control	Scalability	Costwise	Communication with Blockchains	Support and Maturity	SDKs and Tutorials	Total
**Secret**	5	5	4	5	4	5	5	4	**37**
**Oasis**	5	5	4.5	5	4	5	5	4	**37.5**
**Phala**	5	5	4	5	1	5	3	4	**32**
**Integritee**	5	5	5	5	1	5	2	3	**31**
**Ternoa**	5	3	4	5	2.5	5	2.5	2	**29**
**NuCypher**	4	1	4	3	2	2	2	4	**22**
**Lit Protocol**	3	1	5	3	2	2	1	4	**21**
